# INFORMAL CAREGIVING EXPERIENCES: CHALLENGES AND OPPORTUNITIES FOR AN AGE-FRIENDLY UNIVERSITY

**DOI:** 10.1093/geroni/igad104.2837

**Published:** 2023-12-21

**Authors:** Melissa Cannon, C J Johnson, Emily Winters

**Affiliations:** Western Oregon University, Monmouth, Oregon, United States; Western Oregon University, Monmouth, Oregon, United States; Western Oregon University, Monmouth, Oregon, United States

## Abstract

The growing Age-Friendly University (AFU) global network has been helping its members assess their institutions and identify action items for increasing age-friendliness. This research investigates one of the issues identified through previous assessment of an AFU as a weakness and area of opportunity, which is the lack of resources and information for campus community members who identify as informal or unpaid family caregivers. Data from N = 70 campus members (26% faculty, 18% staff, 56% students; 84% female, 16% male) were collected in early 2023 through a campus-wide online survey and analyzed by a team of researchers using quantitative analysis for responses to Likert-type items and thematic analysis for open-ended responses. Issues around physical demands, time demands, financial stress, and social and emotional health while caregiving were assessed. Results indicated that respondents are experiencing different types of stress and need additional support and resources as caregivers, particularly related to respite care, mental health services, financial support, and assistance with physical tasks such as housework and transportation. The findings from this research will be used to develop and share resources around caregiving broadly across the university campus, and to ensure that resources are culturally inclusive, particularly as nearly 25% of the enrollment of undergraduate full-time equivalent students is composed of students from a Hispanic/Latinx background. Findings will also be used to establish community partnerships to harness resources from outside the university, and ultimately to help support the campus community moving forward and to achieve a piece of the university’s AFU vision.

